# Emotional disorder dynamics for patients depending on psychoactive substances at the stages of psychosocial rehabilitation

**DOI:** 10.1192/j.eurpsy.2021.2168

**Published:** 2021-08-13

**Authors:** B. Mykhaylov, O. Kudinova

**Affiliations:** 1 Psychiatry, National Academy of Postgraduate Education, Kiev, Ukraine; 2 Psychotherapy, Kharkiv Medical Academy of Postgraduate Education, Kharkiv, Ukraine

**Keywords:** Psychoactive Substances, Psychosocial rehabilitation, emotional disorders

## Abstract

**Introduction:**

Objective laws of emotional disorder formation, their frequency along with clinic and psychopathological structure have been poorly studied until now.3 groups of patients have been observed: 200 people with alcohol addiction, 180 people with opioid addiction, and 90 people with psychostimulant addiction.

**Objectives:**

All these have influenced our research which goal is to study patients’ emotional state at the stages of psychosocial rehabilitation.

**Methods:**

Signs of psychological and physical addiction, specific personality disorders and decrease in social functioning level have been found for all of the observed patients. Psychodiagnostic research (performed according to Hamilton, Spielberger and Hanin, Buss-Durkee methods) has shown significant increase of depression and anxiety parameters, as well as aggression level for all the patients.

**Results:**

Psychosocial rehabilitation system has been formed and created according to the results of the research. It is built based on a stepwise multimodal principle, including social deprivation, individual and group psychotherapy, craft therapy with an outcome to self-organizing psychotherapeutic groups.

**Conclusions:**

Emotional sphere state normalization occurred during the process of participation in the system. According to catamnesis data of 1 to 2 years, the developed system efficiency is: 72.00 % for patients with alcohol addiction, 64.00 % for patients with opioid addiction, 51.00 % for patients with psychostimulant addiction.

**Disclosure:**

No significant relationships.

